# Supplementary material for: Investigating the dose-response relationship between music and anxiety reduction: A randomized clinical trial
Source: PLOS Ment Health. 2026 Jan 21;3(1):e0000355. doi: 10.1371/journal.pmen.0000355 (PMC12833833; doi:10.1371/journal.pmen.0000355)
Supplement: Attachment — Submitted filename: Dose-Response Response to Reviewers Comments 02-28-2025.docx [file pmen.0000355.s002.docx]

**Manuscript PMEN-D-24-00371**

**Response to Reviewers**

**Deadline: 28 February 2025**

Dear reviewers,

Thank you for your review and for the opportunity to submit a revised draft of “Investigating the dose-response relationship between music and anxiety reduction: A clinical trial” to *PLOS Mental Health*. We are grateful for the time and effort spent doing an in-depth review of our paper and we are eager to share our responses with you. Please see our responses to your comments below.

**Review Comments to the Author**

**Reviewer #1:** *The authors conduct an experiment on the effects of the duration of music listening on reductions in anxiety, with respect to pink noise. While the manuscript is well written, I have concerns about the conclusions made by the authors, given the limitations of the experimental controls used.*

*Major comments:*

*1. The design of the experiment may be insufficient for the conclusions drawn for the paper: where in one condition – ABS + music, in the other condition – pink noise only, with the variable for conditions being the amount of time a participant was exposed to the music stimuli. My main concern is that it does not appear to be a valid control, in that other aspects of music not related to ABS or iso-principles are not controlled for. For example, could the effect be simply an effect of listening to music (as the authors pointed out in the introduction) over pink noise, where the duration is reflective of the calming effects of music? Note that research on mood regulation in music by Saarikallio has pointed out 3 ways that negative emotions (including anxiety) may be downregulated – discharge (or catharsis, which may be what the iso-principle is associated with), solace, and diversion. Any of these could be explaining the significant results observed in the study. A better control would be to have musical elements without either ABS, or iso-principles as part of the composition, as secondary controls. However, this may be a bit too late for this study, given that the design cannot be changed, so this should be made clear in the limitations, and some of the conclusions of the study on the efficacy of ABS and the iso-principle may need to be reduced, in favour of the general effect of music listening.*

*Saarikallio, S. H. (2008). Music in mood regulation: Initial scale development. Musicae scientiae, 12(2), 291-309.*

**Response:** Thank you for this point. In a previous study by Mallik & Russo (2022), it was found that music with ABS was more effective at reducing anxiety than music alone or our control (pink noise). That study was more mechanistic in nature with a design that allowed us to evaluate which aspects of the intervention were effective. By comparison, the current investigation was focused on the “dose” required to achieve reduction in anxiety. To evaluate dose, we needed more conditions than the prior study, which is why we focused on music with ABS as the experimental condition and use of pink noise as the control. Pink noise is commonly used as a control condition in experiments using music listening as the experimental condition, and we agree that it is important to use an active control to avoid there being a placebo effect of having the control condition sit in silence. We have attempted to clarify our motivation for the study in the introduction.

*2. Pink noise appears to exacerbate physiological reactions (nausea)? This could be an additional confound of the study, where participants in the pink noise condition may be experiencing additional negative elements (e.g., boredom, annoyance, nausea) that may be explaining the muted results (see above). While this paradigm may have been a replication of previous work, it still does not offer strong evidence for the effect of ABS on anxiety reduction due to these issues with the experiment (namely with the lack of good controls).*

**Response:** Thank you for raising this point. We believe the one participant that got nauseous was an outlier, as the literature does not suggest nausea to be a common side effect of listening to pink noise. We received no other reports of nausea in the present study, and the previous study also did not report any adverse side effects to listening to pink noise (Mallik & Russo, 2022). Additionally, other literature points out that pink noise can be used to help promote sleep, with a 2022 systematic review of noise and sleep studies finding that 82% of studies utilized pink noise (Capezuit et al., 2022).

*Minor comments:*

*1. Why would listening to pink noise lead to poor adherence in the planned control conditions for 12 and 36 minutes? Can the authors provide more explanation here perhaps?*

**Response:** Thank you for this question. We only observed poor adherence (50%) in the 36-minute condition, which we suspect was due to participant boredom. To balance the design out, we then got rid of the 12-minute condition. This has been clarified in lines 179-185.

*2. May I ask for more information about the auditory stimuli (listening examples)*

**Response:** The auditory stimuli were directly exported from the Vibe app produced by LUCID, which samples tracks from its proprietary music library. The genre of music in this library can broadly be described as ambient instrumental music with embedded nature sounds. Twenty-five music track sequences (playlists) were exported for each duration condition (short, medium, long). The playlists were curated by a recommendation system described in Mallik & Russo (2022) that utilizes an initial mood state specified by the participant. In the current study, the mood state was specified using the self-assessment mannikin rather than the app itself. In this way, the playlists could be pre-generated, meaning that the participant did not need to directly interact with the app. The self-assessment mannikin asks participants to indicate their current level of arousal (5-points) and valence (5-points), resulting in 1 of 25 unique mood states. The number of tracks in a playlist was determined by the duration condition: approximately 3, 6, or 12 tracks for short, medium and long, respectively. All music tracks were embedded with auditory beat stimulation using a method described in Labbé, A. (2025). Device, method, and medium for integrating auditory beat stimulation into music. Patent No. 12,205,566 B2. Washington, DC: U.S. Patent and Trademark Office.

*3. The listening period is quite long for an online study. How did the authors ensure compliance, such as the participants not doing something else while the music was playing.*

**Response:** We asked participants to close their eyes and wear headphones while listening to their assigned audio, in an attempt to ensure they were paying attention to the audio. Because this experiment was conducted entirely online, we had no way to ensure complete compliance, and this is one of the limitations of the study. This has been added to the limitations section of the paper in lines 481-488.

*4. P values cannot = 0, but should be expressed as < .001.*

**Response:** Thank you for pointing this out. We have revised those p values to be < 0.001.

*5. In the discussion (p17), the 3 study aims are mentioned, but only 2 are elaborated on immediately after.*

**Response:** Thank you for this comment. We elaborate on aim 1 in section 4.1 and elaborate on aims 2 and 3 in section 4.2.

*6. The paper lacks a limitations section*

**Response:** Thank you for pointing this out. We have added a limitations section to the paper.

*Reviewer #2: It was a pleasure reviewing this manuscript, which sought to determine if there is an optimal length of time for listening to music with ABS that yields reduced anxiety and improvement in affect for individuals with anxiety. The first goal of the study was to replicate findings from Mallik and Russo (2022) which demonstrated that music with ABS was superior to pink noise control in reducing anxiety, while the second goal was to investigate differences in anxiety and affect based on length of exposure to music and ABS. Below are my comments and recommendations organized according to each of the major sections of the manuscript. Overall, the introduction and results section require minor edits (see below), whereas the methods and discussion require major edits. Comments that may require major edits are indicated by **, which means that either an essential part of the manuscript is absent or that an important part of the manuscript requires explanation or significant revision/editing.*

*INTRODUCTION*

*Overall, the introduction is very clearly written and well-organized. The introduction can benefit from specific discussion of the music and ABS literature as treatment modalities for anxiety.*

*1. The only major anxiety disorder mentioned is generalized anxiety disorder GAD – the introduction can benefit from briefly describing other anxiety disorders particularly in the discussion on different treatment modalities like benzos, SSRIs, CBT, other forms of psychotherapy, etc.*

**Response:** Thank you for raising this. We have added reference to social anxiety disorder and panic disorder in lines 51-54.

*2. Some of the references on the use of medications and CBT are relatively outdated (see reference 6 through 12) – I would suggest making these arguments using the most updated research.*

**Response:** Thank you for pointing this out. We have updated those references to be more recent.

*3. Lines 75-84 (paragraph) reveals the effectiveness of CBT, which is followed by lines 85-94 (paragraph) which appears to abruptly discount everything just mentioned about CBT. Was this intended, or is the intention simply to discuss the pros and cons of this treatment modality?*

**Response:** Thank you for this question. Our intention was to point out the pros and cons of CBT as we are suggesting that listening to music with ABS is a useful complementary method of anxiety management.

*4. Although the introduction does a great job of outlining medications and CBT as treatment modalities for anxiety disorders, it could be improved with a brief discussion on what the literature says about combined medication and psychotherapy as treatment for anxiety disorders.*

**Response:** Thank you for raising this. We have added in discussion about combined medication and psychotherapy as treatment for anxiety disorders in lines 87-90.

*5. Lines 92-95: The wording here proposes a demand for alternative or complementary therapeutic options, and then states that the use of music is one of these alternative approaches. Wording needs to be clarified here as to whether music is considered an alternative or complementary approach – the introduction does make it sound like an alternative approach; however the study design and inclusion criteria suggest otherwise, particularly given that all participants needed to be on medication.*

**Response:** Thank you for pointing this out. We have made some minor changes to the abstract and introduction to highlight how music listening can be complementary with other methods of anxiety management.

*6. Lines 95-108: This paragraph and the subsequent paragraph could be uncovered a little more. For example, were all these studies done on participants using music therapy alone? Did any combine music therapy with other treatment modalities?*

**Response:** Thank you for this question. These sources were talking about music listening, not music therapy, which we have clarified.

*7. Last 2 paragraphs of introduction, lines 160-178: these paragraphs should be in the methods section*

**Response:** Thank you for pointing this out. We have moved those lines to the methods section, lines 198-208.

*METHODS*

*Overall, the methods section requires more organization (e.g. describing processes in chronological order) and clarity of missing information (unclear whether some data were not collected or just left out of the manuscript).*

*Design*

*1. Lines 182-185: how were the exposure times determined for each of the experimental groups?*

**Response:** Thank you for this question. In the prior study on which the present is based (Mallik & Russo, 2022), 24-minute audio interventions were used due to technical limitations, and this was found to be an effective length of time spent listening to music with ABS for anxiety reduction. A meta-analysis on binaural beats (Garcia-Argibay et al., 2019) showed that listening to binaural beats for 20-30 minutes was effective for anxiety reduction. We then determined the 12- and 36-minute exposure times by adjusting the 24-minute audio by 12-minute intervals. We have included this information in lines 173-177.

*2. Line 186-190:*

*a. ** More details are required on the piloting process (e.g. number of pilot participants, how they determined there was poor adherence, etc.)*

**Response:** There were 36 pilot participants. Poor adherence was determined by comparing the amount of time spent completing the survey to the length of the assigned audio (ex: if someone was assigned to a 36-minute audio but only spent 20 minutes on the study, they were considered to have not adhered to the audio). This has been clarified in lines 177-185.

*b. Any thoughts for the discussion section on whether using only the 24-min pink noise impacted the results of the study?*

**Response:** We have addressed this in the limitations section of the paper, lines 495-496.

*3. Line 196: from information presented later in the methods, we learn that participants were randomly assigned to groups once they obtained access to Qualtrics*

*a. Could participants log into Qualtrics on their own time and therefore complete the study whenever? If so, please state this. If not, please include further details.*

**Response:** Yes, participants could log into Qualtrics at any time once they were invited to complete the study. This has been clarified in lines 210-211.

*b. Were participants blind to which group they were assigned? Were the researchers blind to which group participants were assigned? Please clarify.*

**Response:** While the participants were blinded to what condition they were in as well as what all the possible conditions they could have been assigned to were, the researchers were not blinded to the condition. The researchers needed to know which condition each participant was assigned to in order to verify if the participants listened to the entirety of their assigned audio.

*Participants*

*1. ** Inclusion criteria*

*a. Line 206: anxiolytic medication to ensure a clinical diagnosis - justification required here to explain this assumption*

*i. Was a diagnosis for each participant ever confirmed? If so, what was this process and what were the diagnoses?*

**Response:** A diagnosis for each participant was not confirmed. We had the inclusion criteria that participants needed to meet a minimum score on the STICSA as well as needed to be taking an anti-anxiety medication in an attempt to only include participants with clinically significant anxiety. This is a limitation of our study and has been addressed in the limitations section, lines 489-494.

*ii. Was being on an anxiolytic medication confirmed through the Anxiety Coping Methods Questionnaire?*

**Response:** Yes, it was confirmed that participants were taking an anti-anxiety medication through the Anxiety Coping Methods Questionnaire.

*b. For pragmatic purposes, I understand why participants could be on medication, but it is not clear at all why it is part of the inclusion criteria, particularly given the arguments made in the introduction. I am wondering how many of the screened 1310 individuals would have been eligible to participate had they just been on medication, which begs the question – why not include participants who are not on any medication and screen for those who can confirm that they have a diagnosis of a specific anxiety disorder?*

**Response:** Thank you for this question. In an attempt to only include individuals with clinically diagnosed anxiety, participants had to be on an anti-anxiety medication to participate in this study. We were interested in the complementary effects of music listening, not music listening as an alternative to anti-anxiety medication.

*c. More problematic is that, aside from knowing that all participants were on medication, there are no other data on medications (e.g. how long they have taken the medication, whether taken daily or as needed, dose, type of medication, etc.), which makes it impossible to interpret the findings of this study in any other context except for music and ABS, when there are clearly other things to consider that may have impacted the outcomes*

**Response:** Thank you for raising this. As we mentioned in our response to comment B, we were interested in the complementary effects of music listening, not music listening as a stand-alone treatment. While it is unclear how the differences you mentioned would impact the interpretation of the results, the fact that this was a randomized trial makes it unlikely that these additional factors you mentioned could explain the effects seen.

*d. Line 207: moderate trait anxiety – what is the significance of only including participants with a moderate trait anxiety? Justification required here.*

**Response:** The prior study on which the current is based (Mallik & Russo, 2022) found that listening to music with ABS was more effective for anxiety reduction in those with moderate trait anxiety than those with high trait anxiety. This has been explained in lines 224-227.

*2. Recruitment and Screening*

*a. Lines 216-220: this section may read clearer if all recruitment information was moved to the beginning of this section*

**Response:** Thank you for pointing this out. All of the recruitment information was moved to the beginning of the “Participants” section (lines 218-234).

*b. ** Aside from using Prolific, more details are required about the recruitment strategy and screening process*

*c. ** 1310 participants screened*

*i. How were the inclusion criteria communicated to 1310 individuals on Prolific for them know if they were eligible to participate? e.g. how did they know if they were taking any ‘anxiolytic’ medications?*

**Response:** Prolific has a survey that asks about anti-anxiety medication use, and participants were asked again in the pre-screen if they were taking an anti-anxiety medication.

*ii. Did the 1310 individuals need to contact the research team first before being screened? Was screening done online through Prolific? If so, please explain the process in more detail*

**Response:** Prolific screened participants for anti-anxiety medication use, as the study was open to those currently taking an anxiolytic. We did STICSA screening with each participant to identify those with moderate trait anxiety, who were then invited through Prolific to complete the study. This is clarified in lines 283-287.

*iii. Any information on common reasons most of the 1310 were ineligible to participate?*

**Response:** The most common reason for ineligibility was not meeting the threshold for moderate trait anxiety. This has been clarified in lines 231-232.

*3. ** Table 1 - The demographics table only includes gender breakdown and average age for each of the study groups. Throughout the methods section, there are mentions of additional data, but it is unclear if specific data was left out of the manuscript or if the data were not collected throughout the study (e.g. nationality of each participant, type of medication, how long they have been on medication, official diagnoses, etc.). Most of this information provides important context for interpreting the results of this study (which should be included in the discussion). Please include all collected demographic information, whether it is included in Table 1 or in the Participants section.*

**Response:** Thank you for this point. A table has been added that includes type of anxiety medication taken and alternative anxiety management techniques of participants (Tables 2 and 3). We describe the most common nationality amongst the participants in lines 246-247.

*4. Line 226 – more details are required regarding financial compensation*

**Response:** Thank you for raising this. We outlined financial compensation in more detail in our IRB application materials.

*Materials*

*1. Line 232*

*a. What is the full name of the questionnaire used to confirm use of anxiolytic medication?*

**Response:** The questionnaire used was not a specific questionnaire, but rather was a brief questionnaire asking what medication they were using and if they used any alternative methods of anxiety management. This has been clarified in lines 253-257.

*b. Was this questionnaire administered through Qualtrics once participants were given access? If so, how were participants screened for use of medication during the screening process?*

**Response:** Prolific has their own questionnaires in which they ask participants about their medication use. So, participants were first screened by Prolific for anxiolytic use. If they self-identified as using an anti-anxiety medication, they were invited to be screened for our study. Once on Qualtrics for our study, the participants were asked again if they take an anti-anxiety medication. This has been clarified in lines 283-287.

*c. If this questionnaire provided information on other coping methods (e.g. medications, cannabis, alternative techniques, etc.), why not include these data in this study?*

**Response:** This information has been included in Table 3.

*2. Line 239-241*

*a. more details required for Affective Music Recommendation System and SAM, and they were used in Qualtrics together*

**Response:** Please see response to Reviewer 1’s second comment.

*b. I would recommend providing a table (maybe supplementary) with all SAM scores to help the reader understand how each participant was linked to their corresponding playlists*

**Response:** Please see our response to Reviewer 1’s second comment. The 5x5 self-assessment mannikin was used to select a playlist that was pre-generated using the Vibe app. The figures used were taken from a published manuscript that has been cited more than 12,000 times (Bradley & Lang, 1994). The images are copyrighted, and as such, we do not feel they should appear as figures in our paper. If it is felt that we need the images, we can pursue getting the rights but feel this is not necessary as it has been so highly cited.

*3. ** Lines 250-259 – this paragraph requires more clarity re: how the 25 combinations correspond to 25 different playlists, each with a ‘slightly different trajectory’*

**Response:** Please see our response Reviewer 1’s minor second comment.

*4. Line 258 – the SAM was administered after listening to the audio, but only for ‘consistency’? Please explain what this means and why changes in SAM are irrelevant for this study. Has anxiety level ever been studied in the context of SAM scores?*

**Response:** Thank you for raising this. We have run an analysis to compare SAM scores before and after listening to the audio, and the results have been added to the paper in lines 402-417 (also written below).

Short, medium and long music had significant increases in valence compared to the control (pink noise) (Figure 10). Medium music had significant reductions in arousal compared to the control, short music and long music treatments (Figure 11).

Figure 10. Change in valence as measured by SAM (post session minus pre-session). Error bars are standard errors of means. * denotes p < 0.05, ** denotes p < 0.01 by Fisher Randomization, 5000 iterations. Mean ± Standard Deviation Control (Pink Noise): 0 ± 0.96, Short Music: 0.39 ± 0.86, Medium Music: 0.64 ± 0.96, Long Music: 0.59 ± 0.93

Figure 11. Change in arousal as measured by SAM (post session minus pre-session). Error bars are standard errors of means. * denotes p < 0.05, ** denotes p < 0.01 by Fisher Randomization, 5000 iterations. Mean ± Standard Deviation Control (Pink Noise): 0 ± 1.24, Short Music: -0.32 ± 0.85, Medium Music: -0.73 ± 0.91, Long Music: -0.18 ± 1.03

*Procedures*

*1. Order of events needs to be clarified, and should be consistent with timelines presented in all Methods subsections*

**Response:** Thank you for raising this. We have verified timeline consistency across the methods and subsections.

*2. Line 265 - if participants completed the Anxiety Coping Methods Questionnaire and STICSA after obtaining access to Qualtrics, how were they pre-screened to determine that they were on an anxiolytic and had moderate trait anxiety? (same context as above, under Materials, comment 1a.)*

**Response:** Participants were first pre-screened by Prolific, which asks them if they are taking any anti-anxiety medications. After this, we administered the STICSA to participants to ensure they fell into the moderate trait anxiety range. From here, if they met both criteria, they were invited to complete the study. This has been clarified in lines 283-287.

*3. ** Line 273 – Where are the results of asking these questions? If participants were asked to use headphones, did any report not using headphones? Did any participant report being distracted? If so, what did they report?*

**Response:** Out of a total of 144 participants in the study, 49 participants did not wear headphones. We asked the participants on a 3-point scale how distracted they were: 1) Not distracted at all. 2) Yes, I was a little distracted by an interruption but was able to bring my attention back to the listening session. 3) Yes, I was fully distracted by an interruption and was unable to pay any attention to the audio session.

Out of 144 participants, none were fully distracted by interruption, 29 participants were a little distracted by an interruption but were able to bring attention back to the listening session and the rest were not distracted at all.

*RESULTS*

*Overall, the results are well-organized and are presented in a clear and consistent manner.*

*1. I would suggest providing a table for pre- and post- scores from the STICSA and PANAS with score changes, so the reader can view the numbers behind the ‘moderate anxiety trait’ for each study group?*

**Response:** Our data is available on Open Science Framework, including changes in STICSA and PANAS scores.

*2. I would also suggest providing a table for arousal and valence levels from SAM at the start of the study (and post- scores, unless there is justification provided under Materials, comment 4.).*

**Response:** The results of the pre- and post- scores analysis have been added to the results section in lines 402-417.

*3. All figures require a legend or explanation of the asterisks used to represent different significant levels*

**Response:** Thank you for raising this. We have added the explanation of the asterisk to the captions of the figures, and they are also listed here: * denotes p < 0.05, ** denotes p < 0.01, **** denotes p < 0.0001

*4. Figure 4 and 5 – measured by STICSA, or were these evaluated using PANAS?*

**Response:** Thank you for pointing this out. The results displayed in these figures were evaluated using the PANAS. The figure titles have been corrected.

*DISCUSSION*

*Overall, this section requires significant improvement before it can be evaluated for suitability for publication in this journal, as it is missing fundamental components of a discussion section.*

*1. All tables should be introduced in the results section, and not in the discussion.*

**Response:** Thank you for pointing this out. All tables have been moved to the results section.

*2. ** Line 422 – there should be no new data introduced in the discussion section, particularly when this new information should have been reported in the methods/results and would help with proper interpretation of the study findings and results. It is concerning that this information is reported for the first time at the end of the discussion.*

**Response:** Thank you for raising this. A table with the medications participants reported taking (Table 2) has been added to the “participants” section.

*3. ** The following fundamental and essential components are missing from the discussion:*

*a. Interpretation of the results in the context of all data presented in the study*

**Response:** Thank you for raising this. In the context of the data presented in this study, our findings show that listening to music with ABS has the potential to reduce anxiety even further than anti-anxiety medications alone. We do not have data on the dosage of medication that participants were using, which we have pointed out as a limitation in lines 492-494.

*b. Further exploration of difference of findings between current and prior study*

**Response:** The prior and present study participants had similar demographics (age and gender), and all participants had moderate trait anxiety. In the prior study, an increase in positive affect was seen amongst those who listened to music with ABS, whereas in the present study, a decrease in positive affect was seen. This may be due to the fact that in the prior study, participants were allowed to use the app used in the study for up to a month after the study, which may have mitigated any decrease in positive affect seen in the present study. Additionally, the entire experience in the present study was web-based whereas prior study utilized both the web and an app, which made the experience more engaging for participants. Along with this, listening with headphones was required in the prior study while in the present study, we strongly urged participants to use headphones. Because of this, 49 out of the 144 participants in the present study did not use headphones, which theoretically should have reduced the effect of the ABS.

The comparable conditions in the prior study had fewer participants (listening to medium music with ABS had 33 participants in the current study and 22 in the prior study; listening to pink noise had 36 in the present study and 27 in the prior study). As a result, the prior study had slightly less power than the current study. Despite these differences, the results were remarkably similar regarding reductions in anxiety.

*c. Discussion of study findings in the context of existing literature; what is different, what is the same, what does all of this suggest?*

**Response:** This study confirmed the findings shown by Mallik & Russo (2022), i.e. that listening to music with ABS is more effective at reducing anxiety than pink noise. This study also showed that listening to music with ABS significantly reduced negative affect, which was not shown in Mallik & Russo (2022). This all helps strengthen the evidence behind using listening to music with ABS as a complementary method of managing anxiety (in addition to anxiolytic use).

*d. Limitations and strengths of the study*

**Response:** We have added a limitations section to the paper.

*e. What does this study add to the literature and why is it important?*

**Response:** This study adds a better understanding of the dose-response relationship between music listening and anxiety reduction to the literature, as well as helps to validate music with ABS as an evidence-based complementary method for anxiety management. This study demonstrated that varying the amount of time spent listening to music does impact the reduction in anxiety experienced by individuals with clinically significant levels of anxiety. This is important because it demonstrates that music listening can be utilized to immediately reduce anxiety, which would be of interest to individuals coping with anxiety daily. This has been added to lines 475-479.

*f. What are the applications of this study?*

**Response:** The findings of this study can be utilized by individuals coping with anxiety, as this study demonstrated the complementary benefits of music listening to help reduce anxiety in addition to taking an anxiolytic. This study showed that music listening can significantly improve anxiety immediately. This point has been added to the discussion section of the paper in lines 475-479.

**References:**

Bradley, M.M. & Lang, P.J. (1994). Measuring emotion: The self-assessment manikin and the semantic differential. J Behav Ther Exp Psychiatry, 25(1), 49-59. <https://doi.org/10.1016/0005-7916(94)90063-9>

Capezuti, E., Pain, K., Alamag, E., Chen, X., Philibert, V., & Krieger, A. C. (2022). Systematic review: auditory stimulation and sleep. *Journal of Clinical Sleep Medicine*, *18*(6), 1697-1709.

Garcia-Argibay, M., Santed, M. A., & Reales, J. M. (2019). Efficacy of binaural auditory beats in cognition, anxiety, and pain perception: a meta-analysis. *Psychological research*, *83*, 357-372.

Mallik, A. & Russo, F.A. (2022). The effects of music & auditory beat stimulation on anxiety: A randomized clinical trial. PLoS One, 17(3). <https://doi.org/10.1371/journal.pone.0259312>
