# Supplementary material for: Investigating the dose-response relationship between music and anxiety reduction: A randomized clinical trial
Source: PLOS Ment Health. 2026 Jan 21;3(1):e0000355. doi: 10.1371/journal.pmen.0000355 (PMC12833833; doi:10.1371/journal.pmen.0000355)
Supplement: Attachment — Submitted filename: Dose-Response Response to Reviewers Comments 09-21-2025.docx [file pmen.0000355.s003.docx]

**Manuscript PMEN-D-24-00371**

**Response to Reviewers**

**Deadline: 21 September 2025**

Dear reviewers,

Thank you for your review and for the opportunity to submit a revised draft of “Investigating the dose-response relationship between music and anxiety reduction: A clinical trial” to *PLOS Mental Health*. We are grateful for the time and effort spent doing an in-depth review of our paper and we are eager to share our responses with you. Please see our responses to your comments below—all references are listed at the end.

**Review Comments to the Author**

**Reviewer #2:** *The authors aimed to replicate the findings of Mallik and Russo (2022) and to extend this work by examining whether there is a dose-response relationship between listening to music with ABS and its effectiveness in reducing anxiety and improving affect. The manuscript shows significant improvement in its overall organization and now includes the necessary details for a comprehensive and thorough Methods section. Despite these improvements, several concerns remain, which I outline below. While the authors have adequately addressed the original reviewers’ comments, it is important to note that some of the issues I raise below overlap with those earlier concerns. Although the authors responded to them in the rebuttal, these points still need to be addressed directly in the manuscript itself. Below, I provide my comments categorized as MAJOR and MINOR, organized by the primary sections of the manuscript.*

*Minor comments:*

***Abstract*** *– please revise timing of treatment conditions*

**Response:** Thank you for pointing this out. We have revised the timing of the treatment conditions to be accurate.

***Introduction*** *–* *The introduction is well-written, comprehensive, and presents a compelling rationale for the current study and its broader implications. One non-essential suggestion for improvement would be to clarify how anxiety and affect were operationalized, particularly in the following:*

1. ***Line 141*** *– Mallik & Russo (2022), were both anxiety and affect evaluated in the original study? If so, please specify (e.g. STICSA, PANAS, etc.).*

**Response:** Mallik & Russo (2022) also used the STICSA and PANAS for evaluating changes in anxiety and affect, respectively. This has been clarified in lines 215-217 and lines 224-225.

2. ***Line 157*** *– ‘self-reported level of anxiety’, outline how this was operationalized for this study*

**Response:** The study we referenced used the State Trait Anxiety Inventory to determine changes in anxiety. This was clarified in lines 159-162.

3. ***Line 162*** *– specify how ‘anxiety’ and ‘affect’ are being operationalized in the study hypothesis*

**Response:** “Anxiety” and “affect” are operationalized as changes in self-report scores on anxiety and affect scales before and after listening to their assigned audio. This has been clarified in lines 163-166, which can also be found here:

Based on these findings, it was hypothesized that the longer participants listened to music, the greater their reduction in anxiety and improvement in affect would be, operationalized as changes in self-report scores on anxiety and affect scales before and after listening to their assigned audio intervention.

***Methods*** *– The methods section is significantly more organized than the original manuscript and now includes the critical components necessary to evaluate this study for publication. There remain 3-4 areas that are unclear and may benefit from further clarification.*

1. ***Line 174-177*** *– The following sentences remain ambiguous and would benefit from further explanation:*
   a. *The statement that the 24-minute intervention was “found to be an effective length of time” in the prior study is clear; however, the mention of “technical limitations” implies that this duration was chosen due to constraints in study procedures or materials rather than being guided by the literature, empirical evidence, or the research team’s rationale. Please clarify.*

**Response:** The duration of 24-minutes was chosen based on the efficacy demonstrated in the prior study by Mallik & Russo (2022) that deployed the same system. The reference to technical limitations concerns a constraint imposed in the earlier study of each session consisting of 8 tracks. As these tracks were all approximately 3 minutes in length, the overall duration of the session was approximately 24 minutes. For clarity, the mentioning of technical limitations has been removed from the manuscript.

b. *“The 12- and 36-minute exposure times were determined by adjusting the 24-minute audio by 12-minute intervals” remains unclear. It is recommended to include detail from your response to Reviewer #2 (see Methods, Design, 1. Lines 182-185) to improve clarity.*

**Response:** The 12-minute sessions incorporated 4 tracks, and the 36-minute sessions incorporated 12 tracks. As each track was approximately 3-minutes in length, the overall duration was approximately 12-, 24-, or 36-minutes in length. This has been added into the methods section in lines 180-184, which can also be found here:

The 12- and 36-minute exposure times were determined by adjusting the 24-minute audio by 12-minute intervals. The 12-minute sessions incorporated 4 tracks, and the 36-minute sessions incorporated 12 tracks. As each track was approximately 3-minutes in length, the overall duration was approximately 12-, 24-, or 36-minutes in length.

2. ***Line 180 (& Line 249)*** *– The phrase “… comparing the amount of time spent on the study to the length of the assigned audio…” remains unclear. Consider including the example provided in your response to Reviewer #2 (see Methods, Design, 2. Lines 186-190, a.) for greater clarity.*
   a. *Are there any data to illustrate what “poor adherence” looks like (e.g. at what specific time points participants lose focus or stop participating?)*

**Response:** Adherence was determined by comparing the length of the participants assigned audio to the length of time spent on the survey. If the participant spent less time on the survey than the length of their assigned audio, they were considered to have not adhered to the audio, however, we do not have data on the specific time points participants lost focus or stopped participating. This explanation and an example about determining adherence has been added to lines 188-191, which reads as follows:

Adherence was determined by comparing the amount of time spent on the study to the length of the assigned audio. For example, if someone was assigned to a 36-minute audio but only spent 20 minutes on the study, they were considered to have not adhered to the audio.

b. *Was it necessary for the researchers to define an optimal listening duration before concluding that “poor adherence” had occurred? If so, how was this determined? (e.g. both participants A and B are randomly assigned to the 24-minute condition; participant A reports loss of focus or interruption at minute 14, and participate B at minute 16 – is there a difference in their adherence?)*

**Response:** Thank you for this question. We did not differentiate different amounts of adherence in who was included in the study. If the participant spent less time on the survey than the length of their assigned audio, they were considered to have not adhered to the audio.

c. *How were reports of nausea and dizziness collected? Were these reported in real time during the listening task, or only after the audio session concluded? Please specify in the manuscript.*

**Response:** The single report of nausea and dizziness that was received was reported after that participant had completed listening to their audio, at which point they messaged the researchers via Prolific to report the nausea and dizziness. This has been clarified in lines 191-192.

3. ***Line 190*** *– This sentence spears to be incomplete.*

**Response:** Thank you for pointing this out. We have revised this sentence to now read as follows:

Previous meta-analyses on the use of music for anxiety reduction have emphasized the importance of using an active control group to compare the effect of music on anxiety reduction with [35, 45].

4. ***Line 253*** *– “A brief anxiety coping methods questionnaire was used…” Please include the name of the questionnaire here. If the questionnaire is not validated, consider including either the full set of items or a few representative sample questions used to screen participants for anxiolytic medication.*

**Response:** The questionnaire is the same one used in Mallik & Russo (2022). We have included the questionnaire in our resubmission.

5. ***Line 263*** *– If there is an approximate length of time required for participants to complete the SAM used in the AMRS curation process, please specify.*

**Response:** On average, it takes approximately 10 seconds to complete the SAM, and there was no maximum time limit set for completed this scale.

6. ***Line 280*** *– Please elaborate on why administering the SAM after listening was “purely for maintaining consistency”.*

**Response:** Thank you for raising this. The SAM was also administered after participants listened to their assigned audio, and this was initially done purely for maintaining consistency to make the measures administered after listening to their assigned audio mirror those that were administered beforehand, in case there were any order effects. When designing the study, we did not plan for any changes in SAM scores to be considered in the analysis of the data as there were no significant changes in SAM scores reported in the prior study (Mallik & Russo, 2022). However, upon analysis, it was discovered that there were interpretable findings in the changes in SAM scores, which we now include in the results and discussion sections (lines 466-487 and lines 547-565, respectively).

***Results*** *– The results section is comprehensive and clear. The following require minor edits to further improve organization and clarity of findings.*

1. ***Figure 1****- please correct any spelling errors.*

**Response:** Thank you for pointing this out. The spelling errors in the figure have been corrected as seen below.

2. ***Tables 2 & 3*** *– Require detailed titles specifying the source questionnaire and time point (e.g. screening, start of study).*

**Response:** The source questionnaire and time point of administration has been added to Tables 2 and 3.

3. ***Figures*** *– when comparing current to prior study, please indicate length of time for controls.*

**Response:** The length of time for the control has been added to the figure captions.

4. ***Tables*** *– please ensure consistent labeling of control and treatment conditions throughout.*

**Response:** Thank you for catching this. We have consistently labeled the control and treatment conditions throughout the tables.

5. ***Section 3.1 Replication*** *– This section aims to replicate original findings (Figure 2 & 3), however Table 4 & 5 present data from the dose-response investigation, which creates confusion. Please clarify the structure or reorder for consistency.*

**Response:** Tables 4 and 5 are used to show the statistical information for comparisons between the music with ABS groups and the pink noise group in the current study, which was the replication piece. This is why these figures are located under figure 2 and 3 which show changes in anxiety in the previous study compared to the current study.

6. ***Section 3.2 Replication*** *– Similar confusion arises here; Figures 4 & 5 relate to replication of*

*original findings, but Tables 6 & 7 are from the dose-response objective.*

a. *To improve clarity, I would suggest presenting Table 7 (positive affect) before Table 6 (negative affect) to match the order of Figures 4 and 5*

**Response:** Table 6 includes significant comparisons regarding negative affect, including between the music with ABS groups and the pink noise group, which is what figure 4 shows. As there were no significant comparisons regarding positive affect in the replication, but was one comparison that was approaching significance in the extension, table 7 was included later in the manuscript under the extension section.

b. *Table 7 is referred to under Section 3.4, which adds to the confusion. Please revise for clarity.*

**Response:** Table 7 is included in section 3.4 because it is related to the significant findings of the extension of Mallik & Russo as it relates to changes in affect, which is what section 3.4 covers.

7. ***Figures 6-9*** *– Please clarify in the legends that these illustrate treatment group comparisons.*

**Response:** Thank you for your suggestion. We have illustrated the treatment group comparisons in more detail in the figure captions.

8. ***Arousal (Figure 10) and Valence (Figure 11)*** *– these figures present pre-post changes, but Line 280 states that post-listening SAM data were not analyzed. Please resolve this discrepancy.*

**Response:** Thank you for pointing this out. As mentioned in our response to an earlier comment, the SAM was also administered after participants listened to their assigned audio, though this was initially done purely for maintaining consistency to make the measures administered after listening to their assigned audio mirror those that were administered beforehand, in case there were any order effects. When designing the study, we did not plan for any changes in SAM scores to be as not considered in the analysis of the data as there were no significant changes in SAM scores reported in the prior study (Mallik & Russo, 2022). However, upon analysis, it was discovered that there were interpretable findings in the changes in SAM scores, which is discussed in the results section. The manuscript has been updated to discuss these findings in lines 547-565.

***Discussion*** *– The discussion section presents the findings with brief interpretation; however, it would benefit from more in-depth analysis and critical reflection. In particular, the limitations and conclusions sections require substantial revisions before the manuscript can be appropriately evaluated for publication in this journal.*

1. ***Section numbering:*** *The discussion is currently divided into sections 4.1, 4.2, and 4.4, skipping 4.3. Please revise the numbering to ensure proper sequencing and consistency.*

**Response:** Thank you for pointing this out. We have revised the numbers to be accurate.

2. ***Line 419****: The first part of the discussion states there are three aims of the study, yet only two are listed. Please revise to accurately reflect all stated aims or adjust the numbers accordingly.*

**Response:** Thank you for pointing this out. The numbers have been revised to be more clear about the three aims.

3. ***Line 450-454****: This section can be made more concise and should more directly reflect the study’s findings. Consider revising to improve clarity and focus.*

**Response:** Thank you for raising this. We have revised this section, which is now lines 523-534.

4. ***Line 457****: If the authors choose to highlight results that are “approaching significance” (ie., not statistically significant), this should be followed by an appropriate and cautious discussion of their potential implications, avoiding overstatement.*

**Response:** Thank you for raising this. We have added a brief discussion of this finding, which is as follows:

This suggests that it may take longer than 24-minutes for participants to adapt to the music before growing to enjoy the music and hence increase positive affect.

5. ***Line 463****: As the manuscript consistently compares findings relative to the pink noise condition, please maintain this language here as well for clarity and consistency.*

**Response:** Thank you for raising this. The comparison back to pink noise has been added to line 545.

6. ***Lines 475-477****: Please revise this section for clarity and conciseness. Ensure the language reflects only the findings of the current study and avoids broad generalizations not directly supported by the data.*

**Response:** Thank you for raising this. We have expanded upon these lines to now read as follows:

This study provides further support for the contention that music listening can be used to reduce state anxiety in individuals with a clinically significant level of trait anxiety. The findings are of particular relevance in the treatment of individuals coping with anxiety, as this study demonstrated the complementary benefits of music listening to help reduce anxiety in addition to taking an anxiolytic. In other words, these findings encourage the utilization of music listening among those prescribing treatments for anxiety so that music listening is prescribed to help with managing anxiety in addition to taking an anxiolytic to improve treatment outcomes. Music listening may also be prescribed in cases where patients are unable or unwilling to engage in other modalities such as CBT or medication, where other modalities have been ineffective, or where patients have had adverse reactions to other modalities, as engaging in music listening in these scenarios is likely better than the patient receiving no treatment at all.

***Conclusions***

1. *Throughout the manuscript, the authors appropriately describe findings in relation to the pink noise condition. Please ensure this comparative language is used consistently here in the conclusions, to avoid the implication that pink noise functions as a true control.*

**Response:** Thank you for pointing this out. The comparative language back to pink noise has been added to lines 635-636, which now reads as follows:

The findings of the current study suggest that listening to music with ABS is more effective than listening to pink noise in treating state anxiety in people living with moderate trait anxiety.

2. *Some statements in the conclusions are overly broad. Please revise to ensure all claims are grounded specifically in the findings of this study. The first sentence of the paragraph serves as a strong example – concise, accurate, and aligned with the manuscript’s scope. Pease use this as a model for revising the remainder of the conclusion.*

**Response:** Thank you for raising this. We have revised the conclusion to now read as follows:

The findings of the current study suggest that listening to music with ABS is more effective than listening to pink noise in treating state anxiety in people living with moderate trait anxiety. While the expected dose effect was partial with respect to anxiety, we did find clear evidence of a dose effect for negative affect reduction, with longer exposures being better. These findings are important in potentially helping practitioners incorporate music as an alternative to traditional therapeutic channels or part of a stepped-care therapeutic solution for supporting individuals living with anxiety. Further research is needed to better understand the dose effect of music-based digital therapeutics, such as the ideal dose for positive affect improvement, but these findings are encouraging for those with moderate anxiety who are looking an affordable, accessible, and non-medication-based method for relief.

*Major comments:*
**Methods**

1. ***Line 183*** *– While a clear justification is provided for removing the 36-minute condition, the rationale for removing the 12-minute control requires further elaboration, especially in terms of how this adjustment contributes to a “balanced” study design.*

**Response:** Thank you for raising this. Removing the 12-minute pink noise made it so that each condition was being compared to a single control group, rather than the 12-minute and 24-minute music with ABS groups having a control to compare back to and the 36-minute with ABS group not having a control group to compare with (thus, making the study design more “balanced”). This explanation has been added to the manuscript in lines 196-199.

2. ***Line 186*** *– Although a justification is given for using pink noise as the control instead of silence, it would be helpful to explain why pink noise was chosen over other types of noise (e.g. white, brown). The original study used pink noise, but where did this original decision come from?*

**Response:** White noise is considered highest for unease and anxiety provoking effects when compared to pink noise and brown noise (Færøvik, Vikene, & Specht, 2025). Additionally, we made the decision to select pink noise over brown noise as matching acoustic properties is important when selecting a control condition. Pink noise contains all the frequencies that the human ear can hear with a slightly more power in the lower frequencies (3 dB per octave roll off) (Færøvik, Vikene, & Specht, 2025; Shanmugam, 2024). Brown noise has much more power in the lower frequencies at the expense of some of the higher frequencies (6 dB per octave roll off) and is not as close to music in terms of frequency spectra (Færøvik, Vikene, & Specht, 2025; Shanmugam, 2024). Our explanation for using pink noise as the control can be found in lines 200-207 of the manuscript.

**Results**

1. *Control Group - If pink noise is a true control, we should expect no pre-post changes in anxiety or affect. However, we notice that pink noise creates a change on its own, which needs to be discussed fully and consistently (also addressed by Reviewer #1) in the methods and discussion sections.*

**Response:** Pink noise is noise that follows a 1/f energy distribution in the frequency domain (Zhou et al., 2012). Many prior studies involving auditory stimuli have used pink noise as a control stimulus (Garza-Villarreal et al., 2015; Garza-Villarreal et al., 2014; Seifi Ala, Ahmadi-Pajouh, & Nasrabadi, 2018). In addition, the advantage to pink noise as control condition compared to the silent condition is that it helps control for a placebo effect. To this end, pink noise was administered in the same manner as all the other stimuli (i.e., there would be no indication that this was a control). This information regarding the use of pink noise as a control described here can be found in the manuscript in lines 200-207.

**Discussion**

1. ***Positive Affect (Figure 4, Table 6) and Negative Affect (Figure 5, Table 7)****: The observed differences in positive and negative affect should be fully discussed. It is essential to explore how these findings align or diverge from earlier results, and what factors might contribute to these discrepancies.*

**Response:** We have mentioned that the observed differences are likely due to the fact that the prior study required the download and installation of an application which caused some technical difficulties for some participants at the time. The present study had a more seamless participant experience compared to Mallik and Russo (2022), as the prior study required the participant to download and install an application on their phone, and there were technical issues that had to be resolved for some participants. This is somewhat reflected in the data, as the baseline negative affect of participants for the present study was lower than that of Mallik and Russo (2022). We have added this information to the manuscript (lines 510-521).

2. ***Paragraph 437-448****: The authors attribute the failure to replicate findings on positive and negative affect on differences in study design. However, this rationale raises important questions that must be addressed:*
   a. *If study design differences are the primary justification, how did the current study manage to replicate the cognitive and somatic anxiety findings? Are the authors suggesting that design variation affect affect-related outcomes but not anxiety-related ones? This distinction should be clearly articulate and supported.*

**Response:** Thank you for the comment. In response to the prior comment, we mentioned that the present study had a more seamless participant experience compared to Mallik and Russo (2022), as there were many technical issues that had to be resolved for some participants which was somewhat reflected in the baseline negative affect of participants in Mallik and Russo (2022) was higher than the present study. We go into greater detail in the discussion (lines 576-590) and in response to another comment on why this likely impacted affect as opposed to anxiety, arousal, and valence measures.

b. *The mention of design differences (e.g. app-based vs. web-based, headphone requirements) appears for the first time in this section. Given that replication was a stated goal, one would assume that the methodology closely mirrored the original study. Why were these differences not outlined earlier in the manuscript, particularly if they are now being used to explain inconsistencies and are cited as limitations? Clarification is needed regarding why this information was omitted from both the original and revised submissions.*

**Response:** Thank you for raising this. Omitting this information in our prior submissions was an oversight, and the comments raised in this round of revisions helped us to realize the importance of these differences, which we neglected in the earlier write-ups. Information about the differences in the study designs has been added to the methods section in lines 208-213.

3. ***Line 446****: If the “lack of immersion” is being presented as a potential cause of divergence in findings, this has broader implications for the reliability of all study outcomes. If this is an important factor, why did it selectively impact only affect-related measures and not others? Please address this inconsistency or provide a rationale for why ‘immersion’, or lack thereof, would differentially affect certain outcomes.*

**Response:** We have addressed this factor in other responses to comments and in the manuscript in lines 576-590. For your reference, the manuscript reads as follows:

Despite the observed decreases in somatic and cognitive anxiety, reductions in negative affect and arousal, and increases in valence, we observed no significant increases in positive affect. This could be due to several reasons.

The participant population had moderate trait anxiety and were also on anxiolytic medication. Anxiolytic medication such as SSRIs can cause emotional blunting which is indicative of diminished neural processing of both rewarding and aversive stimuli [66], reduced activation in frontal cortex and reward circuits [66, 67], and decreased dopaminergic function in prefrontal regions critical for positive emotions [67]. Outside of anxiolytic effects, given that our participant population had moderate trait anxiety, our participants’ baseline dopaminergic function may have been compromised through chronic stress and anxiety [67]. Other work shows that participants on anxiolytics also have enhanced sensitivity to anxiety-reducing interventions due to their stress response systems being already active [68]. This may have created a situation where music and theta ABS reduced anxiety via parasympathetic activation and theta entrainment without activating the dopaminergic pathways needed for positive affect enhancement [69, 70].

4. ***Lines 455-466****: The results indicating that ABS music listening either reduces or does not change positive affect, while reducing negative affect, are noteworthy but under-discussed. The manuscript would benefit from an interpretation of these results. Consider the following:*
   a. *If these are real effects (ie., not attributable to study design), what are the implications? Why would ABS music reduce anxiety, but not improve positive affect? Does this suggest a specific influence of ABS music on anxiety pathways independent of affect regulation?*

**Response:** Thank you for your comment/suggestion. We provided the following response to this and your additional comments on emotional blunting in the discussion in lines 576-590. The response is also here, for your reference:

Despite the observed decreases in somatic and cognitive anxiety, reductions in negative affect and arousal, and increases in valence, we observed no significant increases in positive affect. This could be due to several reasons.

The participant population had moderate trait anxiety and were also on anxiolytic medication. Anxiolytic medication such as SSRIs can cause emotional blunting which is indicative of diminished neural processing of both rewarding and aversive stimuli [66], reduced activation in frontal cortex and reward circuits [66, 67], and decreased dopaminergic function in prefrontal regions critical for positive emotions [67]. Outside of anxiolytic effects, given that our participant population had moderate trait anxiety, our participants’ baseline dopaminergic function may have been compromised through chronic stress and anxiety [67]. Other work shows that participants on anxiolytics also have enhanced sensitivity to anxiety-reducing interventions due to their stress response systems being already active [68]. This may have created a situation where music and theta ABS reduced anxiety via parasympathetic activation and theta entrainment without activating the dopaminergic pathways needed for positive affect enhancement [69, 70].

5. ***Line 469*** *– If the emotional blunting associated with SSRIS is being proposed as a factor, this needs a more thorough discussion. Specifically:*
   a. *What does the literature say about emotional blunting and its potential role in shaping affective responses in such contexts?*

**Response:** Please see our response above to “Discussion, comment 4.”

b. *If SSRIs are causing blunting, why is there a reduction in negative affect but not a similar change in positive affect? This nuance should be explored in the context of current findings and relevant research.*

**Response:** Please see our response above to “Discussion, comment 4.”

6. ***Figures 10 and 11****: These figures are included in the manuscript but are not addressed in the discussion. Please incorporate a clear explanation of these findings, their relevance to the study objectives, and how they contribute to the interpretation of results.*

**Response:** As mentioned in our response to #8 under results, we included figures 10 and 11 in response to a request made by a different reviewer, though we did not add discussion of these findings as we had no intention to analyze the changes in SAM scores (it was not mentioned in our retrospective registration). As mentioned in our response to earlier comments, we did, however, find interpretable differences in the changes in SAM scores, and have added explanation about these findings to the results and discussion sections in lines 466-487 and lines 549-567.

**Limitations**

1. *Is there any consideration for the implications of conducting the study in participants’ natural and comfortable environments? While this enhances external validity by simulating real-world conditions, it may compromise internal validity due to reduce experimental control (vs. conducting the study in a laboratory or clinical setting). A brief discussion of this trade-off would strengthen the manuscript.*

**Response:** Thank you for raising this. We have added discussion of this trade-off to the limitations section (lines 610-615). The text in the manuscript reads as follows:

Along these lines, while conducting this experiment in participants’ natural environments enhances the external validity of the findings of this study by simulating real-world conditions in which patients with anxiety would be likely to use this intervention, this may have in turn reduced the internal validity of the study as there was less experimental control as compared to conducting the study in a laboratory setting.

2. *Although the study reports statistically significant findings, it would be valuable to consider whether these effects translate into clinically meaningful outcomes. How might these findings impact real-world treatment or intervention strategies?*

**Response:** These findings suggest that music could be “prescribed” as an add-on to traditional methods (i.e. taking an anxiolytic) to improve outcomes for patients coping with anxiety. Additionally, if other treatment modalities such as CBT prove to be ineffective or inaccessible for a patient, or if a patient has adverse reactions to medication, music listening could be prescribed in place of the patient receiving no treatment at all. This discussion has been added to lines 591-601. For your reference, the text in the manuscript reads as follows:

This study provides further support for the contention that music listening can be used to reduce state anxiety in individuals with a clinically significant level of trait anxiety. The findings are of particular relevance in the treatment of individuals coping with anxiety, as this study demonstrated the complementary benefits of music listening to help reduce anxiety in addition to taking an anxiolytic. In other words, these findings encourage the utilization of music listening among those prescribing treatments for anxiety so that music listening is prescribed to help with managing anxiety in addition to taking an anxiolytic to improve treatment outcomes. Music listening may also be prescribed in cases where patients are unable or unwilling to engage in other modalities such as CBT or medication, where other modalities have been ineffective, or where patients have had adverse reactions to other modalities, as engaging in music listening in these scenarios is likely better than the patient receiving no treatment at all.

3. *Consider how access to formal diagnoses might influence the interpretation of results. Would such data refine participant characterization, improve generalizability, or alter the conclusions drawn?*

**Response:** We do not think access to formal diagnoses would necessarily alter the conclusions of the study. Given the reduction in somatic and cognitive anxiety, negative affect, and no significant change in positive affect, this suggests that the main mechanism action of music and theta ABS appears to be parasympathetic nervous system activation.

4. *The lack of information regarding participants’ medication dosages is a notable limitation. Please discuss how access to these variables might have influenced the findings or interpretations.*

**Response:** Because we did not have information on participants’ medication dosage, we cannot say what combination of anti-anxiety medication and music listening is ideal for anxiety reduction. We also do not know the level of emotional blunting that may have been experienced by participants. We have included discussion of these limitations in lines 616-624, which reads as follows:

In an attempt to only include individuals with clinically significant anxiety, participants were required to be taking anti-anxiety medication, which was verified at multiple points during their study participation. However, we did not verify a formal diagnosis of anxiety disorder for each participant. Additionally, no data was collected on the dosage of medications that the participants were taking. Because of this, we cannot say what combination of anti-anxiety medication and music listening is ideal for anxiety reduction. Additionally, because of the lack of dosage information, we cannot speak to the extent of emotional blunting that may have been occurring in the participants, though we suspect that some level of emotional blunting was occurring for many of the participants.

5. *The rationale for using pink noise as the control condition requires further elaboration. Specifically, clarify why this choice represents a limitation, how it may have influenced results, and what alternative control conditions might better isolate the effects of ABS music.*

**Response:** We provided a more elaborate explanation on why we selected pink noise as a control in lines 200-207 of the manuscript, which reads as follows:

Pink noise has a power spectral density proportional to 1/f (frequency)—roughly a 3dB decrease in power per octave—and is known to be present in a range of naturally occurring settings [50], such as in the flow of sand falling in an hourglass and the flow of water in the Nile River [51]. Previous meta-analyses on the use of music for anxiety reduction have emphasized the importance of using an active control group to compare the effect of music on anxiety reduction with [35, 45]. To this end, using pink noise rather than silence helps to control for the possibility of a placebo effect that may be seen when having some groups listen to music while others sit in silence [44].

**Reviewer #3:** *Overall, the manuscript describes very interesting research related to music and the potential as alternative support people with mental health issues. The methods and materials are quite complicated and can be confusing from the way they were described throughout the manuscript. However, previous reviewers have extensively reviewed the theoretical and technical parts of the research which I mostly agree and do not wish to repeat.*

*I just have a few questions/ concerns as the following:*

*1- How were the conditions (short, medium, long) verified from each participants? Since it was administered online, does the application actually measure the actual time of the participants listening to the music? If not and it was only assumed from the group assignment, this may be another limitation.*

**Response:** Thank you for this question. Qualtrics (the platform on which the experiment was conducted) measures how long participants spend on the study. Because this was a single-blind study, we then compared this information to the length of the audio participants were assigned to and determined from here if the participants spent enough time on the survey to have listened to the full audio. We have given an example of this in lines 188-191, which reads as follows:

Adherence was determined by comparing the amount of time spent on the study to the length of the assigned audio. For example, if someone was assigned to a 36-minute audio but only spent 20 minutes on the study, they were considered to have not adhered to the audio.

*2- The results have a lot going on and thus, confusing. Consider explaining in one section under methodology: the statistical tests, pre and post analysis and all the variables (IVs and DVs from questionnaire, SAM, PANAS, STICSA and so on) you're measuring or comparing.*

**Response:** Thank you for your comment. We have re-structured the methods section with updated headings as requested to make things clearer.

*3- Can the effect of ABS also be influenced by participant's factors or situations not measured in the study? Participants reaction to the music may well be influenced by their social status, current life events, works, physical health, the place and time they listen to the music etc. which may temper with the results. This should also be discussed thoroughly.*

**Response:** The effect of binaural beats may be influenced by participant factors. Research shows that ABS stimulation works by entraining brain activity to specific frequencies, but this entrainment is more effective when individuals naturally exhibit oscillations near the stimulating frequency. If endogenous activity at the target frequency (e.g., 4 Hz theta) is absent or weak, the external auditory beat may fail to induce the desired neural synchrony or cognitive/behavioral effect. Other participant-specific factors—such as music preferences—can influence ABS efficacy (Le Scouarnec et al., 2001), which we did not account for in the current study. This text has been added to the limitations section of the manuscript in lines 625-631.

*4- In terms of representation, can the results be generalized to any particular sampling population? I am not familiar with Prolific for the sampling or what areas or countries it covers*

**Response:** On Prolific (the platform from where participants were recruited), it is possible to specify which countries you would like to recruit from. We did not make such specifications; thus, recruitment was open worldwide. Despite this, most participants were from Western countries (USA, Canada, UK, EU), which is stated in lines 324-328.

**Reviewer #4:** *Line 268: "Recruitment was stopped once we reached this minimum sample requirement." However, the flowchart shows that each arm enrolled at least 33 participants, which is over 25% more than the stated minimum of n = 26. Furthermore, the sample sizes across treatment groups are not balanced, ranging from 33 to 41 participants—a difference of more than 20%.*

**Response:** Thank you for catching this. We recruited additional participants above the minimum of 26 to account for the 25-50% participant dropout rate that is present in many online research studies (Galesic, 2006; Hoerger, 2010). Regarding the difference in sample sizes across treatment groups, participants were assigned to one of four groups using the Qualtrics randomizer element. This randomizer, although it attempts to distribute an even number of participants in each treatment group, does not always do so exactly. This was clarified in lines 263-265, which reads as follows:

We recruited additional participants above the minimum of 26 to account for the 25-50% participant dropout rate that is present in many online research studies [52, 53].

*What statistical method was used to calculate the sample size?*

**Response:** We conducted an *a priori* t-test (difference between independent means) power analyses using G-Power 3.1.9. This has been clarified in lines 259-265, which reads as follows:

The sample size was determined by an *a priori* power analysis based on two previous studies that investigated the relationship between binaural beats and anxiety [42, 44]. We conducted an *a priori* t-test (difference between independent means) power analyses using G-Power 3.1.9. The prior studies had a combined Cohen’s D of 0.80, indicating that a minimum of 26 participants per group would be required to achieve a power of 0.80 at a significance of p = 0.05. We recruited additional participants above the minimum of 26 to account for the 25-50% participant dropout rate that is present in many online research studies [52, 53].

*Lines 244–250 would be more appropriately placed in the Results section.*

**Response:** These lines have been moved to the Results section (lines 325-330).

*P-values should be adjusted for multiple comparisons.*

**Response:** Permutation tests are a good way to control the type I error rate for multiple comparisons, it is non-parametric and so makes no assumptions about the underlying distribution of the data that are common in other inferential statistical tests (Camargo, Azuaje, Wang, & Zheng, 2008; Good, 1994; Kuehl, 2000; Önder, 2007). We have added the preceding text to the manuscript (lines 321-323).

*Tables require reformatting:*

*Treatment arms should be presented as columns.*

*For Table 1: add % for gender; show SD for age.*

**Response:** Thank you for catching this. The tables have been updated/reformatted.

*For outcome measures:*

*Show mean (SD) for both baseline and change from baseline.*

**Response:** These have been added to the text after the change from baseline values.

*Avoid displaying unnecessary technical details such as "two-sided", degrees of freedom, etc., in the column headings.*

*Post-hoc power calculations are not meaningful and should be omitted.*

**Response:** We have removed the following columns from the tables as requested: tail of test, degrees of freedom and post hoc power.

**References:**

Bromberg-Martin, E. S., Matsumoto, M., & Hikosaka, O. (2010). Dopamine in motivational control: rewarding, aversive, and alerting. *Neuron, 68*(5), 815-834. <https://doi.org/10.1016/j.neuron.2010.11.022>

Camargo, A., Azuaje, F., Wang, H., & Zheng, H. (2008). Permutation – based statistical tests for multiple hypotheses. *Source Code for Biology and Medicine, 3*(1), 15. <https://doi.org/10.1186/1751-0473-3-15>

Dobrakowski, P., Blaszkiewicz, M., & Skalski, S. (2020). Changes in the Electrical Activity of the Brain in the Alpha and Theta Bands during Prayer and Meditation. *Int J Environ Res Public Health, 17*(24). <https://doi.org/10.3390/ijerph17249567>

Færøvik, U. H., Vikene, K., & Specht, K. (2025). Put the control back in the control condition: are brown, pink, and white noise neutral control stimuli? *Front Neurosci, 19*, 1488682. <https://doi.org/10.3389/fnins.2025.1488682>

Galesic, M. (2006). Dropouts on the web: Effects of interest and burden experienced during an online survey. *Journal of official statistics, 22*(2), 313.

Good, P. (1994). *Permutation tests: A practical guide to resampling methods for testing hypotheses*. New York: Springer Science + Business Media.

Hamilton, J. L., Burke, T. A., Stange, J. P., Kleiman, E. M., Rubenstein, L. M., Scopelliti, K. A., . . . Alloy, L. B. (2017). Trait Affect, Emotion Regulation, and the Generation of Negative and Positive Interpersonal Events. *Behav Ther, 48*(4), 435-447. <https://doi.org/10.1016/j.beth.2017.01.006>

Hoerger, M. (2010). Participant Dropout as a Function of Survey Length in Internet-Mediated University Studies: Implications for Study Design and Voluntary Participation in Psychological Research. *Cyberpsychology, Behavior, and Social Networking, 13*(6), 697-700. <https://doi.org/10.1089/cyber.2009.0445>

Hjorth, O. R., Frick, A., Gingnell, M., Hoppe, J. M., Faria, V., Hultberg, S., . . . Furmark, T. (2021). Expectancy effects on serotonin and dopamine transporters during SSRI treatment of social anxiety disorder: a randomized clinical trial. *Translational Psychiatry, 11*(1), 559. <https://doi.org/10.1038/s41398-021-01682-3>

Kuehl, R. O. (2000). *Design of experiments: Statistical Principles of Research Design* (2nd ed.). Pacific Grove, California: Duxbury Press.

Le Scouarnec, R. P., Poirier, R. M., Owens, J. E., Gauthier, J., Taylor, A. G., & Foresman, P. A. (2001). Use of binaural beat tapes for treatment of anxiety: A pilot study. Altern Ther Health Med, 7(1), 58–63.

Ma, H., Cai, M., & Wang, H. (2021). Emotional Blunting in Patients With Major Depressive Disorder: A Brief Non-systematic Review of Current Research. *Front Psychiatry, 12*, 792960. <https://doi.org/10.3389/fpsyt.2021.792960>

Mallik, A. & Russo, F.A. (2022). The effects of music & auditory beat stimulation on anxiety: A	randomized clinical trial. PLoS One, 17(3). <https://doi.org/10.1371/journal.pone.0259312>

McCabe, C., Mishor, Z., Cowen, P. J., & Harmer, C. J. (2010). Diminished neural processing of aversive and rewarding stimuli during selective serotonin reuptake inhibitor treatment. *Biol Psychiatry, 67*(5), 439-445. <https://doi.org/10.1016/j.biopsych.2009.11.001>

McConnell, P. A., Froeliger, B., Garland, E. L., Ives, J. C., & Sforzo, G. A. (2014). Auditory driving of the autonomic nervous system: Listening to theta-frequency binaural beats post-exercise increases parasympathetic activation and sympathetic withdrawal. *Frontiers in Psychology, 5*(1248). <https://doi.org/10.3389/fpsyg.2014.01248>

Önder, H. (2007). Using Permutation Tests to Reduce Type I and II Errors for Small Ruminant Research. *Journal of Applied Animal Research, 32*(1), 69-72. <https://doi.org/10.1080/09712119.2007.9706849>

Posner, J., Russell, J. A., & Peterson, B. S. (2005). The circumplex model of affect: an integrative approach to affective neuroscience, cognitive development, and psychopathology. *Dev Psychopathol, 17*(3), 715-734. <https://doi.org/10.1017/s0954579405050340>
